# Morphometric changes on the vulva from proestrus to oestrus of nulliparous and multiparous HYPERPROLIFIC sows

**DOI:** 10.1111/rda.14178

**Published:** 2022-06-20

**Authors:** Paloma De la Cruz‐Vigo, Alvaro Rodriguez‐Boñal, Angel Rodriguez‐Bonilla, Alejandro Córdova‐Izquierdo, Sonia S. Pérez Garnelo, Ernesto Gómez‐Fidalgo, Mercedes Martín‐Lluch, Raúl Sánchez‐Sánchez

**Affiliations:** ^1^ Department of Animal Reproduction National Institute for Agricultural and Food Research and Technology (INIA‐CSIC) Madrid Spain; ^2^ Livestock Feed COSAD Toledo Spain; ^3^ Autonomous Metropolitan University Xochimilco Unit México City Mexico

**Keywords:** gilt, oestrus, sow, vulva size

## Abstract

The aim of this study was to assess whether vulvar morphometric changes occurring in female pigs during proestrus and oestrus could be objective, accurate and predictive indicators of the onset to oestrus and thus performed artificial inseminations at the most appropriate time. For that purpose, pictures of vulvas from 60 hyperprolific females (30 gilts and 30 sows) during proestrus and oestrus were taken once a day. Vulva measurements (area, perimeter, length and width) on these pictures were performed using the image processing ImageJ software. Gilts and sows showed statistical differences (*p* < .01) in all vulvar morphometric measurements between proestrus and oestrus. Statistical differences in vulvar metrics were detected 24 h before the onset to oestrus, affecting all vulvar measurements in gilts, whereas only vulvar width was affected in sows. The image analysis used in this study may contribute to the development of smart technology in swine farming.

## 1 INTRODUCTION

Artificial insemination (AI) is the reproductive biotechnology most widely used in pig production systems. However, determining the optimal time to inseminate still relies on a subjective evaluation of behavioural and physical signs of the sow to detect the onset of oestrus by technicians. Sexual behaviour of the sows during oestrus is affected by oestrogen concentration and is characterized by a variety of signals, such as vocalizations, riding of pen mates when group‐housed, changes in physical activity and/or feed intake decrease (Soede & Kemp, 1997). However, the most important signal is immobilization or ‘standing’ in response to back pressure from a teaser boar, another gilt or sow, or from a person (Soede & Kemp, 1997; Steverink et al., 1999; Simoes et al., 2014; Weng, 2020). Moreover, during oestrus the vulva appears hyperaemic, congested and swollen with a marked increase in its size (Soede & Kemp, 1997; Romoser et al., 2020).

In pigs, variability among females in the duration of oestrus and as consequence, in the time of ovulation after the onset of oestrus, is seen. There are many factors which influence this variability such as housing conditions, level of stress, season, parity, genotype, boar stimulation and so on, but in general, oestrus lasts from 45 to 60 h and is relatively constant within a farm (Steverink et al., 1999). Ovulation is considered to occur at the beginning of the last third of oestrus. Duration of oestrus varies between sows and gilts and thus, in mature sows, oestrus can last 2–3 days whereas in gilts 1–2 days (Steverink et al., 1999; Soede & Kemp, 1997). The development of automated systems that allows for improvements in reproductive efficiency, such as the use of image analysis for oestrus detection, can be an important contribution towards achieving today's and leading ‘smart swine farming’ (Mahfuz et al., 2022). Thus, the aim of this study was to analyse vulvar morphometric variations on hyperprolific gilts and sows during the periovulatory period in order to establish a relationship between these morphometric fluctuations and the onset of oestrus.

## 2 MATERIALS AND METHODS

### 2.1 Animals

The study was carried out in a commercial farm on 60 females (30 sows and 30 gilts) from a hyperprolific DanBred hybrid line (Landrace × Large White). All animals recruited to this study were reared in a conventional production system following Spanish and European livestock and welfare regulations.

### 2.2 Vulvar morphometric measurements

Pictures of the vulvas were taken once a day at the same time each day (from 8:30 to 10:30 h AM) during proestrus and oestrus phases using a digital camera (Canon EOS 700D; Canon Inc.). Oestrus detection was performed twice a day, and the time in which sows showed the standing reflex in the presence of a teaser boar was considered as the onset of oestrus. For gilts, the study covered the period between the last day of oestrus synchronization treatment (20 mg/d of altrenogest fed for 18 consecutive days) and the end of oestrus signs. In sows, pictures were taken from the weaning to the end of oestrus signals. All pictures were taken at the same distance from the back of the sow (50–60 cm), and a ruler was placed next to the vulva as reference in order to establish the scale.

### 2.3 Image analysis with ImageJ software

Picture analysis was carried out using the public domain image processing program ImageJ v 1.52a (National Institute of Health and the Laboratory for Optical and Computational Instrumentation; Rueden et al., 2017).

**FIGURE 1:**
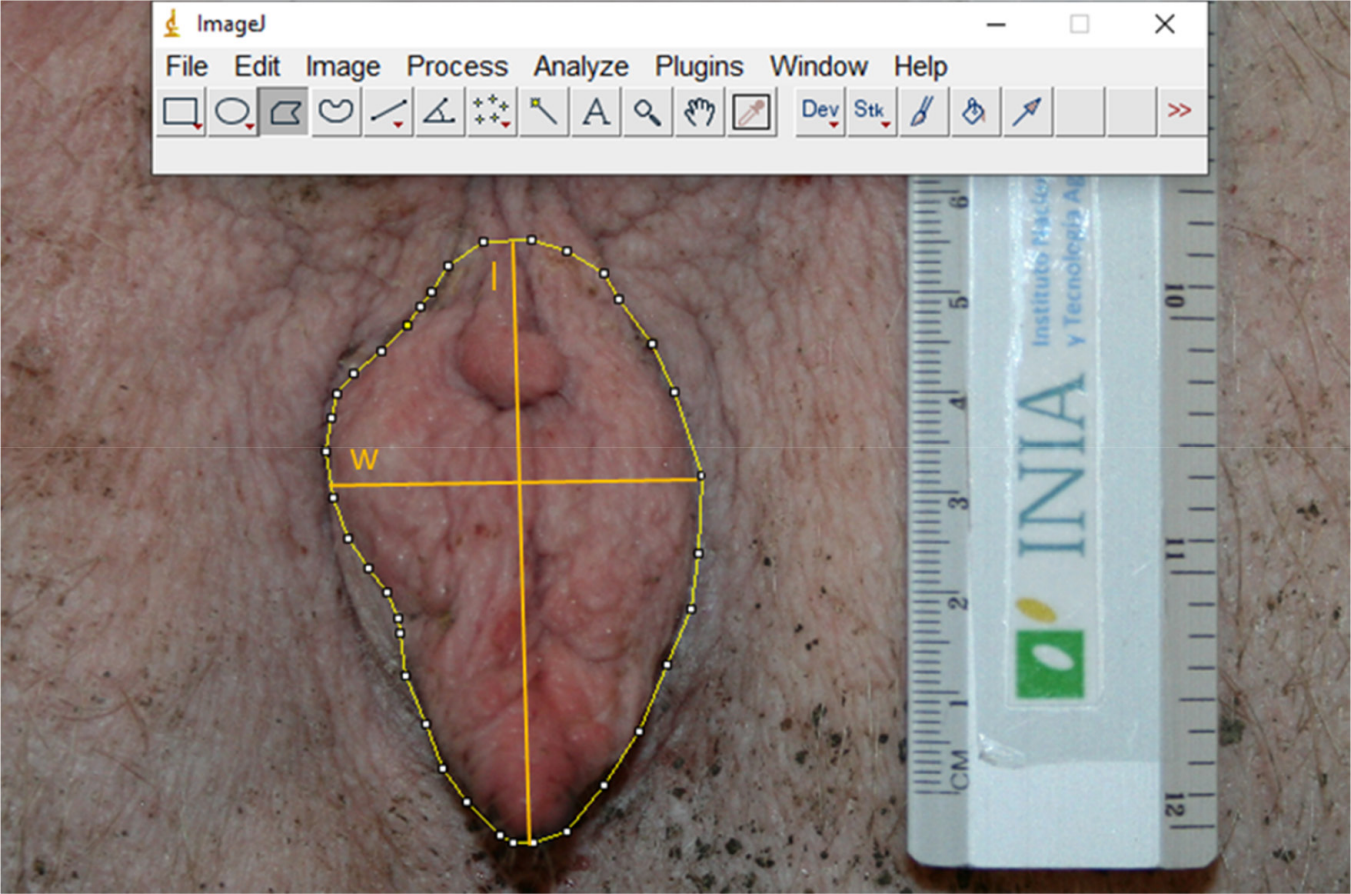
Vulvar metric parameters measured with image J on a multiparous sow: Area (cm^2^), perimeter (cm), vulva length (l; cm) and vulva width (w: cm)

The measurements made on the images of the vulva of each sow were vulvar area (cm^2^), perimeter (cm), vulva length (cm) and vulva width (cm) as shown in Figure 1. Vulva metrics were entered and stored in a database.

### 2.4 Statistical analysis of morphometric data

The statistical study of the differences between the mean vulvar measurements was performed on the data obtained from the analysis of images from 144 h before oestrus to 24 h after the onset of oestrus in gilts and from 96 h before oestrus to 72 h after the onset of oestrus for sows.

The collected data of vulva measurements were analysed using SAS 9.1 software (SAS Institute Inc., 1990). Means between proestrus and oestrus phases in both groups of sows were compared using a linear model by means of the least square means test (LS means). Data are presented as the means of each group and the standard error of the mean (SEM). Significance was set at *p* < .05.

## 3 RESULTS

Vulva measurements for nulliparous and multiparous sows during proestrus and oestrus are shown in Table 1. For gilts, statistical significance between proestrus and oestrus in all the measures studied were found. Thus, the differences between the means of the two reproductive phases were 3.30 ± 0.46 cm^2^ for vulvar area (*p* < .0001), 1.52 ± 0.24 cm for perimeter (*p* < .0001), 0.51 ± 0.09 cm for vulva length (*p* < .0001) and 0.49 ± 0.07 cm for vulva width (*p* < .0001) (Table 1). In addition, statistical differences (*p* < .05) were found between the 24 h before the oestrus and the 24 h after with respect to the previous period (from 144 to 44 h before oestrus) in all measurements (Table 2).

**TABLE 1:** Vulvar morphometric measurements during proestrus and oestrus of nulliparous (*n* = 30) and multiparous (*n* = 30) sows (mean ± SEM)

		PROESTRUS	ESTRUS	*p*
AREA (cm^2^)	GILTS	12.12 ± 0.21	15.43 ± 0.41	<.0001
	SOWS	16.94 ± 0.51	19.99 ± 0.76	.001
PERIMETER (cm)	GILTS	12.87 ± 0.11	14.39 ± 0.21	<.0001
	SOWS	16.02 ± 0.23	17.43 ± 0.34	.0008
VULVA LENGTH	GILTS	4.49 ± 0.04	5.00 ± 0.08	<.0001
	SOWS	5.67 ± 0.10	6.13 ± 0.15	.0091
VULVA WIDTH (cm)	GILTS	3.40 ± 0.03	3.89 ± 0.06	<.0001
	SOWS	3.77 ± 0.05	4.16 ± 0.08	<.0001

*Note*: Means between rows are significantly different (column *p*).

**TABLE 2:** Vulvar morphometric measurements in gilts (*n* = 30) during proestrus and oestrus (mean ± SEM)

Hours related to oestrus	Vulva area (cm^2^)	Vulva perimeter (cm)	Vulva length (cm)	Vulva width (cm)
−144	10.98 ± 0.55^c^	12.35 ± 0.29^c^	4.29 ± 0.11^cd^	3.24 ± 0.08^c^
−120	11.03 ± 0.51^c^	12.29 ± 0.26^c^	4.22 ± 0.10^d^	3.31 ± 0.08^c^
−96	11.73 ± 0.51^c^	12.78 ± 0.26^bc^	4.47 ± 0.10^bcd^	3.34 ± 0.08^bc^
−72	12.04 ± 0.53^bc^	12.91 ± 0.27^bc^	4.58 ± 0.10^bc^	3.32 ± 0.08^c^
−48	13.07 ± 0.51^b^	13.33 ± 0.26^b^	4.63 ± 0.10^b^	3.54 ± 0.08^b^
−24	15.09 ± 0.51^a^	14.25 ± 0.26^a^	4.99 ± 0.10^a^	3.79 ± 0.08^a^
0 → oestrus	15.77 ± 0.52^a^	14.52 ± 0.27^a^	5.07 ± 0.10^a^	3.92 ± 0.08^a^
24	15.49 ± 0.52^a^	14.48 ± 0.27^a^	4.96 ± 0.10^a^	3.92 ± 0.08^a^

*Note*: ^a–d^Means within a column without a common superscript letter differ (*p* < .05).

For sows, means of vulvar measurements between oestrus and days before (proestrus) showed statistical differences for all parameters too. In this group of females, differences between proestrus and oestrus means were 3.05 ± 0.92 cm^2^ for area (*p* = .001), 1.41 ± 0.41 cm for perimeter (*p* < .001), 0.47 ± 0.18 cm for the longitudinal axis (vulva length; *p* < .01) and 0.39 ± 0.09 cm for vulva width (*p* < .0001) (Table 1). This group also showed significant differences (*p* < .05) from 24 h before oestrus when compared with the previous period on vulvar width (Table 3).

**TABLE 3:** Vulvar morphometric measurements in sows (*n* = 30) during proestrus and oestrus (mean ± SEM)

Hours related to oestrus	Vulva area (cm^2^)	Vulva perimeter (cm)	Vulva length (cm)	Vulva width (cm)
−96	14.43 ± 1.05^bc^	14.72 ± 0.47^d^	5.16 ± 0.20^d^	3.55 ± 0.11^d^
−72	15.61 ± 1.03^bc^	15.42 ± 0.46^cd^	5.43 ± 0.20^cd^	3.61 ± 0.11^cd^
−48	17.09 ± 1.03^abc^	16.15 ± 0.46^bc^	5.75 ± 0.20^bc^	3.78 ± 0.11^bc^
−24	19.98 ± 1.03^a^	17.38 ± 0.46^ab^	6.16 ± 0.20^ab^	4.12 ± 0.11^a^
0 → oestrus	21.35 ± 1.09^a^	18.00 ± 0.49^a^	6.46 ± 0.21^a^	4.16 ± 0.11^a^
24	20.70 ± 1.21^a^	17.65 ± 0.54^a^	6.19 ± 0.24^ab^	4.26 ± 0.13^a^
48	18.45 ± 1.03^ab^	16.91 ± 0.46^ab^	5.90 ± 0.20^abc^	4.02 ± 0.11^ab^
72	19.73 ± 1.11^a^	17.46 ± 0.50^ab^	6.14 ± 0.22^ab^	4.11 ± 0.11^a^

*Note*: ^a–d^Means within a column without a common superscript letter differ (*p* < .05).

## 4 DISCUSSION

In recent years, a large number of methods to accurately predict the onset of oestrus under field conditions have been developed and thus, previous studies have used infrared thermography to detect changes in vulvar skin temperatures, devices to detect changes on electrical resistance of the vaginal mucus and the ultrasonography to detect or predict the occurrence of ovulation (Weng, 2020; Simoes et al., 2014; Hidalgo et al., 2014; Williams & Luzbel de la Sota et al., 2017).

Studies of the morphometric changes of the vulva have also been carried out both to establish a correlation with gilts puberty (Graves et al., 2020) and to identify gilts with a higher reproductive potential (Romoser et al., 2020; Mills et al., 2020), but not to relate these changes to onset of oestrus as in our case. In both, gilts and sows, the differences between proestrus and oestrus on vulvar metrics were significant in all measurements. In addition, we detected that morphometric variations of the vulva were detected from 24 hours before the onset of oestrus and maintained throughout oestrus. In gilts, it affected all vulvar measurements, whereas in multiparous sows, only the vulva width was affected.

The use of modern sensor technologies, such as 3D cameras, to detect indicators of animal growth, health, behaviour and welfare has proven useful in contributing to the development of a ‘precision livestock farming’ (D'Eath et al., 2018; Mahfuz et al., 2022). A remote monitoring tool can provide accurate information and ensure economic benefits of the farm (Mahfuz et al., 2022). In this context, the results of this study show that image analysis of the vulva can be an useful tool to detect the onset of oestrus in gilts and sows. This research study opens a way for the development of a novel method for detecting the onset of oestrus under field conditions, allowing inseminations to be performed at the most appropriate moment and contributing to the development of a smart swine farming.

## CONFLICT OF INTEREST

None of the authors have any conflict of interest to declare.

## AUTHOR CONTRIBUTIONS

Conceptualization Raúl S.S.; Methodology Raúl S.S., Paloma C.V., Sonia P.G., Mercedes M.L.,Ernesto G.F., Alvaro R.B., Angel R.B.; Resources Raúl S.S., Alvaro R.B., Angel R.B.; Analysis of data Paloma C.V., Raúl S.S., Ernesto G.F., Sonia P.G.; Review Alejandro C.I., Sonia P.G.; Writing‐original draft Paloma D.V., Raúl S.S., Mercedes M.L.; Writing and editing Sonia P.G.

## Data Availability

The data that support the findings of this study are available from the corresponding author upon reasonable request.
